# Mineral dust aerosols promote the formation of toxic nitropolycyclic aromatic compounds

**DOI:** 10.1038/srep24427

**Published:** 2016-04-14

**Authors:** Takayuki Kameda, Eri Azumi, Aki Fukushima, Ning Tang, Atsushi Matsuki, Yuta Kamiya, Akira Toriba, Kazuichi Hayakawa

**Affiliations:** 1 Graduate School of Energy Science, Kyoto University, Yoshida-Honmachi, Sakyo-ku, Kyoto 606-8501, Japan; 2 Institute of Medical, Pharmaceutical and Health Sciences, Kanazawa University, Kakuma-machi, Kanazawa, Ishikawa 920-1192, Japan; 3 Institute of Nature and Environmental Technology, Kanazawa University, Kakuma-machi, Kanazawa, Ishikawa 920-1192, Japan

## Abstract

Atmospheric nitrated polycyclic aromatic hydrocarbons (NPAHs), which have been shown to have adverse health effects such as carcinogenicity, are formed in part through nitration reactions of their parent polycyclic aromatic hydrocarbons (PAHs) in the atmosphere. However, little is known about heterogeneous nitration rates of PAHs by gaseous NO_2_ on natural mineral substrates, such as desert dust aerosols. Herein by employing kinetic experiments using a flow reactor and surface analysis by Fourier transform infrared spectroscopy with pyridine adsorption, we demonstrate that the reaction is accelerated on acidic surfaces of mineral dust, particularly on those of clay minerals. In support of this finding, we show that levels of ambient particle-associated NPAHs in Beijing, China, significantly increased during heavy dust storms. These results suggest that mineral dust surface reactions are an unrecognized source of toxic organic chemicals in the atmosphere and that they enhance the toxicity of mineral dust aerosols in urban environments.

Nitrated polycyclic aromatic hydrocarbons (NPAHs) are a major class of toxic compounds found in ambient airborne particulates^1^,^2^. NPAHs are produced from chemical reactions of polycyclic aromatic hydrocarbons (PAHs) in the atmosphere^3^,^4^,^5^ as well as from anthropogenic sources such as fuel combustion^6^,^7^. Some types of NPAHs are formed via gas-phase reactions of semi-volatile PAHs, and are subsequently deposited on airborne particulates. For example, 2-nitropyrene is formed from the gas-phase reaction of pyrene (Py) with OH radicals in the presence of NO_2_^3^, and 2-nitrofluoranthene is formed via OH or NO_3_ radical-initiated reactions in the gas-phase^3^. One of the most abundant NPAHs is 1-nitropyrene (1-NP), which is formed through the combustion of fossil fuels such as coal and diesel fuel^6^,^7^. 1-NP, which is considered a probable carcinogen^8^, can also be formed from gas-particle phase heterogeneous reactions^9^,^10^,^11^,^12^,^13^,^14^,^15^. It is formed by the reaction of Py with gaseous NO_2_ on various substrates such as graphite (as a model of soot)^9^ and a variety of metal oxides (as models of mineral aerosols)^11^,^12^,^14^,^15^. However, heterogeneous formation of atmospheric 1-NP has previously been thought to be negligible because the reaction rate and the yield of 1-NP through this process are not sufficient to account for ambient 1-NP concentration^10^,^13^,^14^,^16^. Previous studies of heterogeneous NPAH formation used simple inorganic oxides such as SiO_2_, Al_2_O_3_, and TiO_2_ as models of mineral dust aerosols^11^,^15^,^17^, but these substances lack the complexity of real mineral dust aerosols and thus may not be good models for investigating heterogeneous NPAH formation. Mineral dust is a major component of airborne particulates on a global scale^18^. It is transported by winds from deserts or semiarid regions^19^, which account for 40% of the total world land area^20^. Organic compounds adsorbed on the surface of mineral dust can have important health implications^21^. However, heterogeneous nitration of PAHs by gaseous NO_2_ on natural mineral substrates such as desert dust aerosols has not yet been examined.

We hypothesized that the heterogeneous formations of NPAHs on mineral dust could be more important than previously thought because natural mineral aerosols could have more reactive surface than the model materials previously used. To test this hypothesis, we examined the effects of (*i*) authentic mineral dust on the formation of 1-NP from Py and NO_2_ and (*ii*) heavy dust storms on ambient particle-associated 1-NP in Beijing, China. The results of both studies indicate that mineral dust aerosols dramatically increase the conversion of Py to toxic 1-NP.

## Results and Discussion

### NO_2_ exposure experiments of particle-bound Py

**Figure 1:**
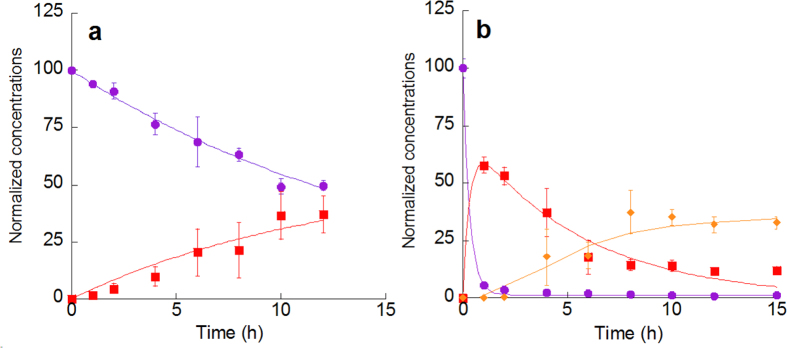
Concentrations of Py and nitropyrenes (1-NP and DNPs) on quartz (**a**) and CDD (**b**) (expressed as a percent of the initial Py concentration) after exposure to 3 ppmv NO_2_ for the indicated times. The data points represent mean values (±1 SD) of triplicate experiments: circles, Py; squares, 1-NP; diamonds, DNPs (=1,3-DNP + 1,6-DNP + 1,8-DNP). The curves for Py decay are exponential nonlinear least-squares fits assuming first-order reactions. See Methods for details. The curves for nitropyrene formation are for illustrative purposes only.

Degradation of Py was measured under 3 ppmv NO_2_-air in the dark. On quartz (SiO_2_) particles, Py was slowly converted to 1-NP, reaching a ~40% yield in 12 h (Fig. 1a). On Chinese desert dust (CDD) particles, more than 90% of the initial amount of Py was degraded and the maximum yield of 1-NP was attained after a reaction time of 1 h (Fig. 1b). 1-NP then gradually converted to dinitropyrenes (DNPs) (Fig. 1b). Py was undetectable after 15 h, indicating that the Py coated on CDD was completely consumed. Other mononitropyrene isomers were not detected. Desert dust is generally composed of various minerals such as quartz, corundum (α-Al_2_O_3_), clay minerals, carbonates, feldspars, and hematite (Fe_2_O_3_)^22^. To determine which components contribute to rapid nitration, we compared the percentage of degraded Py (*D*_Py_) and the yield of 1-NP (*Y*_1-NP_) during a reaction time of 2 h on various substrates that generally constitute desert dust. The most active components were natural montmorillonites (referred to herein as montmorillonites A and B), kaolin, and saponite as well as Arizona Test Dust (ATD; standard test dust made from Arizona desert sand) and CDD (Table 1). In most of these cases, the conversion of Py to 1-NP was completed within 2 h (Table 1 and Supplementary Fig. S1). DNP formation was observed except on saponite. Kaolin, montmorillonites A and B and saponite are types of clay minerals. X-ray diffraction (XRD) analyses showed that ATD and CDD also contain clay minerals (Supplementary Fig. S2). For the other mineral substrates, such as quartz, carbonates (limestone and dolomite), and feldspars, *D*_Py_ and *Y*_1-NP_ were less than 20%, and no DNP was formed during the NO_2_ exposure (Table 1, Supplementary Fig. S1).

To quantify the rate of degradation of Py on each substrate, the kinetics of the heterogeneous reaction between NO_2_ and Py adsorbed on the substrates tested in this study were determined by following the consumption of Py as a function of NO_2_ exposure time. We found that the rate of degradation of Py on each substrate could be fitted to a pseudo-first-order exponential function (see Methods) using nonlinear least-squares fitting. The apparent rate constants of the pseudo-first-order reaction, *k*_obs_, were 2.9 × 10^−4^ – 2.5 × 10^−3 ^s^−1^ on CDD, ATD, and clay minerals and 2.5 × 10^−6^ − 9.0 × 10^−5 ^s^−1^ on the other substrates when the concentration of NO_2_ was 3 ppmv. The *k*_obs_ values and the corresponding apparent reaction probabilities (*γ*), which are defined as the fraction of collisions between NO_2_ gas molecules and the surface-adsorbed Py molecules that leads to reactive loss of Py, are summarized in Table 1. For a simple bimolecular reaction mechanism, in which the rate of the surface reaction is expressed with an apparent first order rate constant *k*_obs_, the value of *γ* is calculated as follows^16^,^23^,^24^:


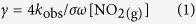


where *σ* represents the effective cross section of a Py molecule (~0.8 nm^2^)^16^, *ω* is the mean thermal velocity of NO_2_, and [NO_2(g)_] is the gas-phase NO_2_ concentration. Previous studies have shown that the observed rate for the heterogeneous reaction of PAHs with gaseous reactants such as NO_2_ and O_3_ exhibits no significant dependence on the initial surface coverage of PAHs (*θ*_PAH,0_) if *θ*_PAH,0_ is less than 1, i.e., submonolayer regime^23^,^24^,^25^. In order to eliminate the effect of the fractional surface coverage of Py (*θ*_Py,0_) on reactivity, therefore, the amount of Py per unit gram of the substrates was controlled in this study so that the estimated *θ*_Py,0_ did not exceed unity (Table 1). The *k*_obs_ and *γ* values on the clays were typically two orders of magnitude larger than those on the other substrates. The obtained kinetic parameters for the reaction on graphite (*k*_obs_ = 1.9 × 10^−5 ^s^−1^, *γ* = 3.2 × 10^−9^), which was used as a control support material, were in the range of previously reported values obtained on typical carbonaceous particles^9^,^13^,^26^,^27^. The reactivity of Py on CDD (*k*_obs_ = 8.6 × 10^−4 ^s^−1^, *γ* = 1.4 × 10^−7^) was considerably greater than that on graphite, although their specific surface areas are similar (~20 m^2 ^g^−1^). Therefore, under our experimental conditions, the difference in the heterogeneous reactivity of Py on the different substrates is not attributable to a difference in the initial surface coverage of Py.

### Surface acid property of substrates

Clay minerals are made up of layered aluminosilicates, which consist of tetrahedral silicate (T) and octahedral aluminate (O) sheets^28^. Clays are classified according to the relative number of T and O layers. For example, kaolinite consists of alternating O and T layers (OT structure). In montmorillonite, in contrast, one O layer is sandwiched between two T layers (TOT structure). The tetrahedral cation Si^4+^ can be replaced by Al^3+^ or Fe^3+^ and the octahedral cation Al^3+^ by Mg^2+^ or Fe^2+^. This internal substitution of cations by cations of lower valence results in a deficiency of positive charge. To balance the charge, cations, which are generally exchangeable, are introduced between the layers^29^. Clay minerals generally exhibit Brønsted and/or Lewis acidity^28^,^29^. Brønsted acidity is attributable to the presence of interlayer exchangeable cations. These cations polarize coordinated water molecules and induce their dissociation into ions. Therefore, the strength of an acid site depends on the type of interlayer cations that are present^29^. In contrast, Lewis acidity is derived from electron-accepting sites in the structures, i.e. the interlayer transition-metal ions within the silicate structure and Al^3+^ ions exposed at crystal edges^29^.

**Figure 2:**
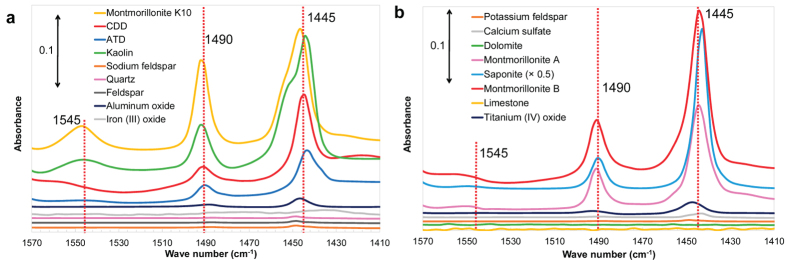
IR spectra of pyridine adsorbed on the mineral substrates examined in this study. To improve legibility, the data were split into two panels.

Nitration of PAHs is catalysed by acid^30^. In fact, several studies have shown that gaseous acids such as HNO_3_ and HCl enhanced the rate of the heterogeneous nitration of PAHs by NO_2_^10^,^15^. Thus, the surface acid property on mineral dust may play a role in the heterogeneous nitration of Py. The surface acid properties of solid catalysts, including clay minerals, can be examined using Fourier transform infrared spectroscopy (FT-IR) with pyridine as a probe^31^,^32^. When pyridine binds to Brønsted acid sites, pyridinium ions are produced, which have an absorption band around 1545 cm^−1^. In contrast, pyridine molecules coordinated to Lewis acid sites have an absorption band around 1445 cm^−1^. The band at 1490 cm^−1^ is attributed to both molecules. The spectra of pyridine adsorbed onto some substrates (CDD, ATD, montmorillonites, kaolin, and saponite) have absorption bands at 1445 cm^−1^ and 1490 cm^−1^, while no absorption band is observed around 1545 cm^−1^, except in the cases of kaolin and montmorillonite K10 (Fig. 2). This suggests that CDD and ATD, as well as clay minerals, have abundant acid sites, particularly Lewis acid sites. On the contrary, the spectra of the other substrates displayed no clear peaks, indicating that they have no or few acid sites on their surface. The largest *k*_obs_ value was obtained for the reaction on montmorillonite K10, an acid-activated clay. Additionally, pre-titration of the surface acid sites of ATD with gaseous NH_3_ (see Methods) significantly inhibited the reaction of Py with 3 ppmv NO_2_, i.e., *k*_obs_ was reduced by approximately 60% and no DNP was formed during the 12 h reaction period (Table 1 and Supplementary Fig. S1). These results strongly suggest that substrates showing acidic surface properties have an accelerating effect on the rate of heterogeneous nitration of PAHs by NO_2_.

### Possible mechanisms for nitration of Py on mineral dust

**Figure 3:**
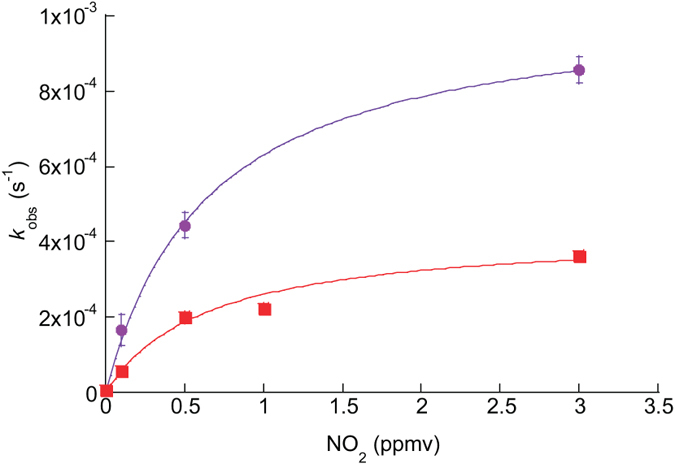
Pseudo-first order rate coefficient (*k*_obs_) as a function of gas-phase NO_2_ concentration. The curves are nonlinear least-squares fits based on Langmuir–Hinshelwood-type mechanism (equation (2)). The upper data set was for CDD, the lower one for ATD. The error bars represent one standard error derived from nonlinear least-squares fitting for the Py decay plots.

Gaseous N_2_O_4_ in equilibrium with gas-phase NO_2_ plays a key role in NO_2_ heterogeneous chemistry^33^. The gaseous N_2_O_4_ would adsorb to the surface of substrates. Several researchers proposed a PAH nitration mechanism in which electrophilic reagents such as N_2_O_4_H^+^ are formed from N_2_O_4_ under acidic conditions^15^,^34^. The electrophiles then attack aromatic compounds to form the corresponding nitrated compounds^15^,^34^. In the case where the N_2_O_4_-induced mechanism contributes to the rapid nitration of Py, the quadratic dependence of the formation of N_2_O_4_ on the NO_2_ concentration would affect the relationship between the gas-phase NO_2_ concentration and the observed decay rate of Py. The values of *k*_obs_ increased nonlinearly with an increase in the gaseous NO_2_ concentration for both CDD and ATD (Fig. 3, Supplementary Table S1), which is in agreement with previous studies^35^, and indicates that the reaction is governed by the Langmuir–Hinshelwood-type mechanism. In the Langmuir–Hinshelwood model, a gas-phase reactant is partitioned between the gas-phase and the surface, and the reaction takes place either between the adsorbed reactant and another surface-bound reactant or between the adsorbed reactant and the surface itself. When nitration takes place *via* the reaction of Py with surface-adsorbed NO_2_ that is in equilibrium with gas-phase NO_2_, the relationship between *k*_obs_ and the gas-phase NO_2_ concentration can be modeled using the following equation:


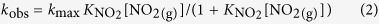


where *K*_NO_2__ is the NO_2_ gas-to-surface equilibrium constant and *k*_max_ is the maximum rate coefficient that would be observed at high NO_2_ concentrations. Figure 3 shows fitting curves based on equation (2) using a nonlinear least-squares fitting. The fitting parameters *k*_max_ and *K*_NO_2__ are listed in Supplementary Table S1. The relationship between the obtained values of *k*_obs_ and [NO_2(g)_] is reasonably simulated by equation (2) over the examined range of [NO_2(g)_] values. This indicates that the reaction order varies between different ranges of [NO_2(g)_] values. That is, the reaction is described by first-order kinetics in NO_2_ at low values of [NO_2(g)_] (i.e., *K*_NO_2__[_NO_2_(g)_] ≪ 1). In contrast, the reaction is described by zeroth-order kinetics in NO_2_ at high values of [NO_2(g)_] (i.e., *K*_NO_2__[_NO_2_(g)_] ≫ 1). This relationship has been widely reported for the reaction between surface-bound PAHs and NO_2_^16^^,^^35^. When nitration is attributed to surface-adsorbed N_2_O_4_ in equilibrium with gaseous N_2_O_4_, in contrast, equation (2) can be modified as follows:


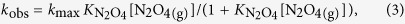


where *K*_N_2_O_4__ is the N_2_O_4_ gas-to-surface equilibrium constant and [N_2_O_4(g)_] is the gas-phase N_2_O_4_ concentration. Equation (3) can be further modified as


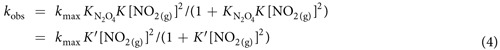


where *K* is the equilibrium constant for the following reaction:


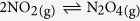


and *K*′ is the product of *K* and *K*_N_2_O_4__. As shown by the fitting curves based on equation (4) (Supplementary Fig. S3), this model does not seem adequate for simulating the relationship between *k*_obs_ and [NO_2(g)_], in particular for ranges of low [NO_2(g)_] values. That is, when [NO_2(g)_] is 100 ppbv, simulated values of *k*_obs_ based on equation (4) are significantly lower than the values of *k*_obs_ obtained from experiments. Therefore, surface-adsorbed N_2_O_4_ in equilibrium with gaseous N_2_O_4_ is unlikely to be effective for the nitration of Py on the dust surface, at least in the range of low [NO_2(g)_] values (i.e., atmospheric concentration levels). Finlayson-Pitts pointed out that gaseous N_2_O_4_ in equilibrium with gas-phase NO_2_ could be ruled out as a key intermediate in the heterogeneous chemistry of NO_2_ at low NO_2_ concentrations^36^, which supports our conclusions. She also suggested a possible contribution of the asymmetric dimer ONONO_2_, which could be formed by a direct reaction of surface-adsorbed NO_2_ with gaseous NO_2_, to the heterogeneous chemistry of NO_2_^36^. The importance of this “directly formed” asymmetric dimer for the nitration of surface-bound PAHs is currently unclear and difficult to discuss at this stage.

Lewis acid sites on alminosilicates are proposed to function as electron acceptors, leading to the formation of aromatic radical cations *via* electron transfer^28^,^29^. The radical cations of several kinds of PAHs, such as Py, perylene, anthracene, and benzo[*a*]pyrene, which form on the surface of aluminosilicates, have been identified by spectroscopic methods, such as electron spin resonance (ESR)^37^. These cations would couple with the surface NO_2_ to yield NPAHs^28^, similar to the nitrous acid-catalysed (NAC) nitration mechanism^38^. That is, the rate-determining step would be the subsequent addition of NO_2_ to the aromatic radical cation yielding a σ–complex (Wheland intermediate), and the deprotonation of this complex would constitute the final fast step which produces the nitrocompound (Supplementary Fig. S4). Thus, our finding that the Lewis acid property of the substrates probably plays a role in nitration (see the previous section), suggests that the rapid formation of 1-NP on mineral dust is the result of NO_2_ reacting with the radical cations of Py, which form on the surface Lewis acid sites (Supplementary Fig. S4). Pöschl *et al*. proposed a theoretical framework, termed the Pöschl–Rudich–Ammann (PRA) framework, for aerosol surface chemistry and gas–particle interactions^39^. Previous studies have successfully employed the PRA framework to reproduce experimental results for the aerosol surface reactions of PAHs with gaseous species^16^. In the PRA framework, the gas–particle interface is divided into a gas phase, a particle bulk (substrate), and two monomolecular layers, i.e., a quasi-static surface layer consisting of non volatile particle components (e.g., Py) and a sorption layer consisting of adsorbed volatile molecules (e.g., NO_2_). The particle bulk can interact with the quasi-static surface layer *via* electron donor–acceptor and charge-transfer interactions and influence the chemical properties of the quasi-static surface layer and related kinetic parameters^39^. Therefore, the heterogeneous chemistry that we propose, in which a quasi-static surface layer consisting of Py is activated by Lewis acid sites on the dust particle bulk followed by nitration by a sorption layer of NO_2_, can be reasonably described by the PRA framework (Supplementary Fig. S4).

### Atmospheric implications

Typical atmospheric concentrations of NO_2_ at major cities around the world are of the order of several tens of ppbv^40^, which are lower than the concentrations that we used in the NO_2_ exposure experiments. The value of *k*_obs_ for the Py degradation on CDD under 50 ppbv NO_2_ is predicted to be (6.7 ± 5.5)×10^−5 ^s^−1^ from equation (2), and the corresponding value of *γ* is (7.3 ± 6.0) × 10^−7^ (errors represent one standard error). Thus, the lifetime of the CDD particle-bound Py is calculated to be 4.1 h, i.e., the apparent reaction rate of Py with NO_2_ on desert dust can compete with the reaction rate of Py with OH radicals in the gas-phase^3^, which is believed to the dominant process by which Py is lost in the atmosphere^3^. The efficiency of the gas-phase OH-initiated nitration is quite low, as the total yield of nitropyrenes is less than 1%^3^. On the other hand, the high yield of nitro compounds *via* this heterogeneous process (Table 1) may result in a high concentration of atmospheric NPAHs.

**Figure 4:**
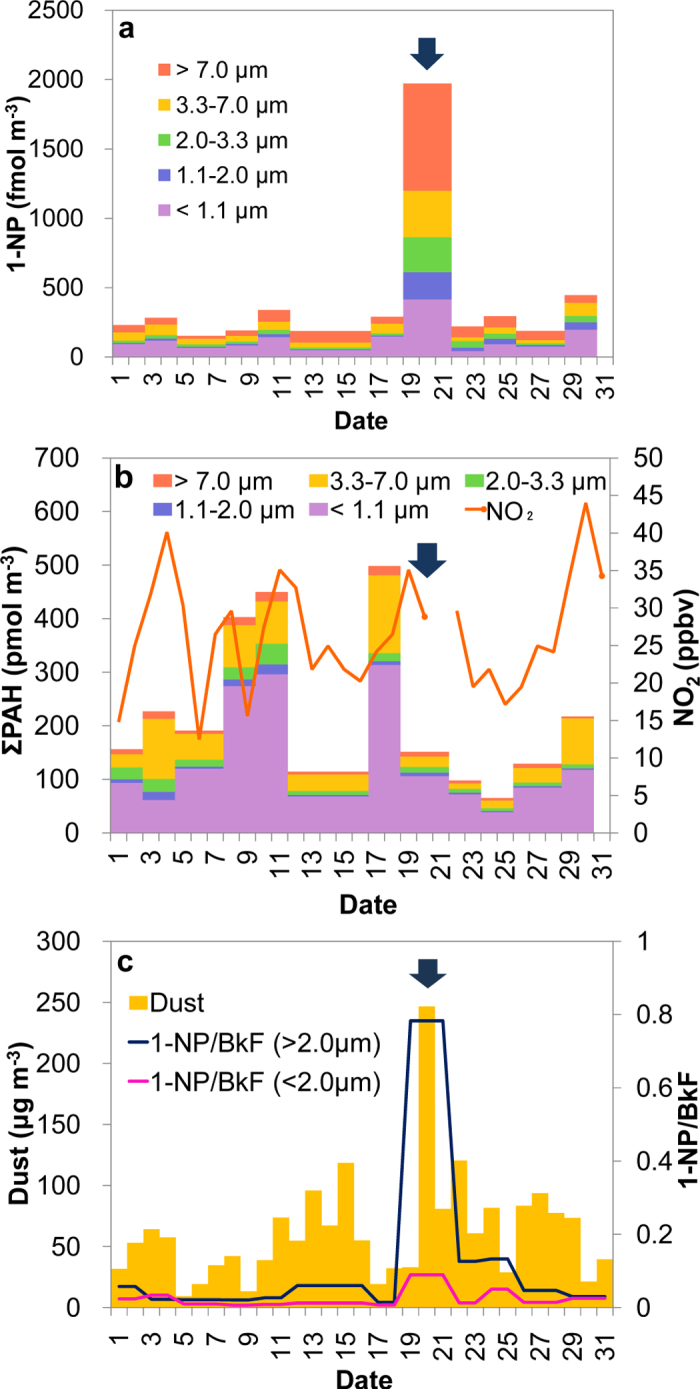
Atmospheric dust, PAHs, NO_2_ and 1-NP concentrations in Beijing in March 2010. (**a**) Size-fractionated particle-bound 1-NP. (**b**) Gaseous NO_2_ and size-fractionated particle-bound PAHs. (**c**) Aeolian dust. Variation in concentration of 1-NP relative to that of BkF (1-NP/BkF) is also shown in (**c**). The daily mean concentrations of aeolian dust were obtained from the LIDAR DSS Observation Data Page^52^. The NO_2_ data was converted from the daily API value obtained from the website of the Beijing Public Net for Environmental Protection^53^. Arrows indicate a heavy dust period.

During the month of March, 2010, we measured the concentrations of particle-bound PAHs and particle-bound 1-NP and obtained data on the concentrations of gaseous NO_2_ and aeolian dust in Beijing. On 20 March 2010, when a heavy dust storm hit Beijing^41^, the concentration of particle-bound 1-NP was considerably higher than that during non- or low-dust periods (Fig. 4a), although concentrations of NO_2_ and PAHs were not unusually different (Fig. 4b). Anthropogenic emission processes such as fossil fuel combustion are regarded as the dominant sources of 1-NP and PAHs^6^,^7^. To determine whether 1-NP was secondarily formed on the dust particles, we evaluated the concentration of 1-NP relative to that of benzo[*k*]fluoranthene (BkF), a fairly unreactive and non-volatile PAH^10^. If sources of these compounds do not change, then the 1-NP/BkF ratio should not change (assuming negligible differences in the degradation rates of them). However, the 1-NP/BkF ratio considerably increased during the period of heavy dust, particularly in the coarse fractions of particle diameters (>2.0 μm) which mainly contain the natural mineral dust^42^ (Fig. 4c). Similar increases in the ratio were observed during heavy dust periods in April and May, 2011 (Supplementary Fig. S5). The 1-NP/BkF ratios during the heavy dust periods significantly differed from those during non-/low-dust periods (*p* < 0.05, Mann-Whitney U test). Atmospheric PAHs can adsorb to mineral dust particles when the dust plumes pass over polluted regions^21^. Aluminosilicates such as clay minerals strongly adsorb PAHs as a result of their Lewis acid properties^43^. Thus, a considerable fraction of the particle-bound 1-NP during the heavy desert dust episodes was likely formed on the dust particles. Surface-adsorbed Py is expected to have formed a submonolayer on the ambient dust particles because the fractional surface coverage of Py on the dust particles, which was estimated from the typical surface area of dust particles in Beijing^44^ and the observed atmospheric concentrations of dust and Py, was less than unity (see Supplementary Results). Therefore, the nitration mechanism proposed in this study (Supplementary Fig. S4) would also be applicable to the heterogeneous nitration of Py on the ambient dust particles.

1-NP might be formed in part from a night-time reaction of dust-bound Py with gaseous N_2_O_5_^45^. However, the heavy dust storm on 20 March, 2010, was observed from 6:00 to 14:00^46^, i.e., in the presence of sunlight. This indicates that, for this duration, the N_2_O_5_ reaction had negligible impact on the atmospheric formation of the dust-bound 1-NP, because NO_3_ in equilibrium with N_2_O_5_ is rapidly photolyzed during the day^10^. The reactive uptake of NO_2_ on illuminated Py, which depends on the intensity of light, also leads to the formation of a trace amount of 1-NP^47^. A reaction of photoexcited Py with surface-adsorbed NO_2_ has been proposed as a nitration mechanism, in which radical cations of Py are expected to form, as in the case of the dark reaction on the surface of dust particles. Thus, the formation of 1-NP on dust particles may be enhanced in the day and the proposed photo-enhanced mechanism may have partly contributed to the formation of 1-NP during the dust storm in March 2010. In contrast, the 1-NP/BkF ratio increased during 11–12 May 2011, when a heavy dust storm was observed in the night (from 20:00 on 11 May to 2:00 on 12 May)^46^. This indicates that the nitration of Py on dust particles is efficiently promoted even under dark conditions. Although HNO_3_ might also participate in the nitration of Py, our kinetics results showed that Py was more readily nitrated by NO_2_ than by HNO_3_ (see Supplementary Fig. S6 and Supplementary Results). Therefore, the reaction of Py with HNO_3_ appears to have little influence on the formation of 1-NP on dust particles. Under the sampling conditions we employed, we cannot rule out the possibility that some of the dust-bound 1-NP was formed on the quartz fiber filters used for sample collection. Furthermore, we cannot rule out the possibility that some of the 1-NP was formed on the ground. That is, some dust particles might have been temporarily deposited on the ground, where the reaction might have occurred, and then resuspended in the air. Although our results clearly show that mineral dust aerosols efficiently catalyse the heterogeneous nitration of PAHs and thus could be an unrecognized source of NPAHs in the environment, further investigation is required to elucidate the details of where the reaction occurs.

Several PAH derivatives, including 1-NP, have been shown to induce cytotoxic or inflammatory responses in respiratory and immune cells^48^. Damage to airway epithelial cells and pro-inflammatory responses are key events in the invasion and recognition of inhaled allergens^49^. Thus, the heterogeneous formation of PAH derivatives on mineral dust aerosols could contribute to respiratory problems such as asthma. Interestingly, the surface soil of several Japanese cities has been found to be largely contaminated with DNPs, which are powerful direct-acting mutagens^50^. DNPs may be formed on the soil surface through catalytic nitration of their parent Py or 1-NP with nitrogen oxides, because significant amounts of DNPs were found to be formed on clay minerals, which are major components of soil, when Py was exposed to NO_2_ (Supplementary Fig. S1). Thus, the catalytic nitration of PAHs on natural minerals needs to be considered as a source of environmental NPAHs. To completely understand the factors affecting the formation rate of the dust-bound NPAHs, e.g., relative humidity and solar radiation intensity, detailed kinetic experiments and further observation of ambient NPAHs are required.

## Methods

### Heterogeneous reaction of Py with NO_2_

Py was initially added to the substrates at a ratio of ~1 nmol mg^−1^. At this concentration, we estimate that the surface coverage of Py was less than 0.7 for all the substrates (Table 1). Hence, Py was regarded as monolayered in all the reactions assuming a uniform adsorption. Py was heterogeneously reacted with various concentrations of gaseous NO_2_/air in a Pyrex flow reactor under constant reaction conditions at 298 ± 1 K and <2% relative humidity in the dark (Supplementary Fig. S7). The reaction products and the residual Py after the prescribed reaction time (normally 1–12 h) were extracted with dichloromethane. The extracted chemicals were identified and quantified by gas chromatographic-mass spectrometric (GC/MS) analysis.

### Curve fitting

In order to quantitatively evaluate the rate of degradation of Py on each substrate, the kinetics of the heterogeneous reaction between NO_2_ and Py adsorbed on the substrates tested in this study were determined by following the decay of Py as a function of NO_2_ exposure time. The degradation of Py showed an exponential pattern, suggesting that the reactions are reasonably described by pseudo-first-order kinetics. In our experiments, the reactions of Py adsorbed on some substrates were not complete, finally reaching a plateau independent of NO_2_ exposure time (e.g., Montmorillonite B in Supplementary Fig. S1). In such cases, the experimental data were fitted by a plateau-shifted first-order exponential function as shown in equation (5)^12^:


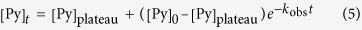


where [Py]_*t*_ is the concentration of adsorbed Py at a given time, [Py]_0_ is the initial concentration of adsorbed Py, [Py]_plateau_ is the concentration of adsorbed Py of the plateau, and *k*_obs_ is the apparent rate constant of the pseudo-first-order reaction. In equation (5), both *k*_obs_ and [Py]_plateau_ are fitting parameters. In the case with no plateau, the experimental data were fitted with a simple first-order exponential function.

### Field measurements

Airborne particulates were collected on the roof of a five-story building approximately 20 m above ground level at the Research Center for Eco-Environmental Sciences, Chinese Academy of Sciences (116.34° E, 40.01° N, Beijing, China) during 1–31 March 2010 and 25 April–30 May 2011, which periods included heavy dust storms^41^,^51^. The sampling site is located in Northern Beijing and is primarily a residential and commercial area, where dominant PAH sources include vehicular traffic and fuel combustion for cooking and/or heating. Samples were collected with a high-volume five-stage cascade impactor (Andersen air sampler; SIBATA, AH-600F) on quartz fiber filters at a flow rate of 566 L min^−1^. The collection periods were usually 2–3 days per sample (Supplementary Table S2). The filter samples were stored at 253 K until subjected to analysis. The airborne particulate samples were pretreated as described in Supplementary Methods. Subsequently, PAHs and 1-NP in the sample solutions were quantified by high-performance liquid chromatography (HPLC) (Supplementary Methods).

Concentrations of aeolian dust measured at the Sino-Japan Friendship Centre for Environmental Protection in Beijing (116.43° E, 39.99° N) in March 2010 were obtained from LIDAR (Light Detection and Ranging) DSS (Dust and Sandstorm) Observation Data Page provided by the Ministry of the Environment, Government of Japan^52^. Daily concentrations of atmospheric NO_2_ and particulate matter smaller than 10 μm (PM_10_) were obtained as Air Pollution Index (API) which were posted on the website of the Beijing Public Net for Environmental Protection^53^. The obtained API values, i.e., mean concentrations measured at 12 observation sites in Beijing, were converted to volume fraction or mass concentration according to the previous report^41^. Since the LIDAR data at Beijing in 2011 were not available, we show the concentration of PM_10_ as a substitute of the dust concentration (Supplementary Fig. S5).

### Characterization of substrates

The surface acid property of the substrates were evaluated with FT-IR by using pyridine as a probe. The substrate sample was pressed into a 10 mg of wafer having a surface area of ca. 0.8 cm^2^ on each face, and mounted into the quartz IR cell with CaF_2_ windows. The sample disk was evacuated at 573 K for 1 h, followed by the adsorption of pyridine vapor at 373 K for 5 min and further evacuation at 423 K for 1 h. The pyridine adsorption infrared spectra were recorded on a Cary-670 FT-IR spectrometer (Agilent Technologies) accumulating 256 scans in the 4000 to 400 cm^−1^ wavenumber range at a resolution 4 cm^−1^. The background spectra were collected prior to the adsorption of pyridine and subtracted from the sample spectra.

According to the previous report^54^, the X-ray diffraction (XRD) patterns of ATD and CDD were recorded from 2 to 65° 2θ every 0.02° 2θ on a Rigaku D8 Ultima IV diffractometer with CuKα radiation using a generator voltage of 40 kV and a generator current of 30 mA. Chemical composition of CDD was determined with an X-ray fluorescent spectrometer (Rigaku, Simultix 12) according to Japanese Industrial Standard (JIS) R2216 “Methods for X-ray fluorescence spectrometric analysis of refractory products”. Obtained results are shown in Supplementary Fig. S2 and Supplementary Table S3. The specific surface area and the size distribution of the substrates were measured on the basis of multi point BET method (Beckman Coulter, SA3100) and the Coulter principle (Beckman Coulter, Multisizer 3), respectively. Obtained results are shown in Supplementary Table S4. The initial surface coverages of Py, *θ*_Py,0_, on the substrates were calculated using the effective cross section of a Py molecule and the obtained BET surface areas of substrates (Table 1).

### Materials

ATD (ISO 12103-1, A2 Fine Test Dust) was obtained from Power Technology. Pre-titration of the surface acid sites of ATD with gaseous NH_3_ was performed as follows: an aliquot of the ATD sample was heated at 573 K for 1 h under a He flow at 50 mL min^−1^, followed by the adsorption of NH_3_ at 373 K by passing 0.5% NH_3_/He through the ATD at a flow rate of 100 mL min^−1^ for 1 h and further He exposure at 373 K for 0.5 h in order to remove residual NH_3_ from the surface of the sample. Quartz and graphite were purchased from Kanto Chemical. Titanium (IV) oxide (NMIJ RM 5711-a) was obtained from National Institute of Advanced Industrial Science and Technology (AIST), Japan. Montmorillonites A and B (JCSS3102 and JCSS3101, respectively) and synthesized saponite (JCSS3501) were obtained from the Clay Science Society of Japan. Kaolin (JCRM R605), sodium feldspar (JCRM R702), and potassium feldspar (JCRM R703) were obtained from the Ceramic Society of Japan. Dolomite (JDo-1), feldspar (JF-1), and limestone (JLs-1) were obtained from Geological Survey of Japan. Calcium sulfate (99.993% metals basis) and aluminum oxide (α-phase, 99.95% metals basis) were purchased from Alfa Aesar. Iron (III) oxide (99.999%-Fe) was obtained from Strem Chemicals. Montmorillonite K10 was purchased from Sigma-Aldrich. CDD was collected from Kumtagh Desert in China (94.45° E, 40.00° N) at a depth of ca. 10 cm. The sieved CDD (<0.38 μm) was sterilized by dry heat at 453 K for 2 h.

### Statistical analysis

Results of the heterogeneous reactions are expressed as mean from three independent experiments ± standard deviation (SD). Nonlinear least-squares curve fitting based on the Levenberg-Marquardt algorithm was performed using KaleidaGraph 4.5 J (Hulinks). The errors of the fitting parameters represent one standard error values. The significance of the difference of 1-NP/BkF ratios was evaluated by Mann-Whitney U test. A value of *p* < 0.05 was considered to be significant.

## Additional Information

**How to cite this article**: Kameda, T. *et al*. Mineral dust aerosols promote the formation of toxic nitropolycyclic aromatic compounds. *Sci. Rep*. **6**, 24427; doi: 10.1038/srep24427 (2016).

## Supplementary Material

Supplementary Information

## Figures and Tables

**Table 1:** Observed pseudo-first order rate constants for the reaction of Py on the substrates examined in this study with 3 ppmv NO_2_ (***k***_obs_), apparent reaction probabilities of NO_2_ with the surface-adsorbed Py (*γ*), percentage of degraded Py (***D***_Py_), yields of 1-NP (***Y***_1−NP_), and initial surface coverages of Py (***θ***_Py,0_).

Substrates	*k*_obs_ × 10^5^ (s^−1^)*	*γ* × 10^8^*	*D*_Py_ (%)†	*Y*_1−NP_ (%)†	DNP formation‡	*θ*_Py,0_ × 10^2^
Chinese desert dust (CDD)	86 ± 4	14 ± 1	96	53	+	2.8
Arizona test dust (ATD)	36 ± 1	6.1 ± 0.2	88	58	+	7.7
Kaolin§	110 ± 10	18 ± 1	98	60	+	1.5
Montmorillonite A	53 ± 5	9.0 ± 0.8	95	89	+	2.0
Montmorillonite B	29 ± 4	4.9 ± 0.7	84	79	+	5.8
Saponite	39 ± 3	6.6 ± 0.4	82	73	−	0.27
Potassium feldspar	1.1 ± 0.2	0.19 ± 0.03	14	10	−	11
Sodium feldspar	0.30 ± 0.06	0.05 ± 0.01	12	6	−	58
Feldspar	0.86 ± 0.14	0.15 ± 0.02	17	4	−	27
Limestone	1.4 ± 0.1	0.24 ± 0.01	18	5	−	21
Dolomite	0.83 ± 0.15	0.14 ± 0.03	16	4	−	11
Calcium sulfate	1.5 ± 0.5	0.25 ± 0.09	6	0	−	49
Quartz	1.7 ± 0.1	0.28 ± 0.01	9	5	−	63
Aluminum oxide	0.25 ± 0.00	0.04 ± 0.00	2	1	−	6.7
Iron (III) oxide	9.0 ± 3.3	1.5 ± 0.6	17	0	−	6.9
Titanium (IV) oxide	1.4 ± 0.0	0.24 ± 0.00	14	3	−	5.2
Montmorillonite K10||	250 ± 20	43 ± 3	100	6	+	0.72
ATD w/ NH_3_ titration¶	15 ± 2	2.5 ± 0.4	62	31	−	7.7
Graphite#	1.9 ± 0.1	0.32 ± 0.01	9	1	−	2.6

^*^Errors represent one standard error derived from nonlinear least-squares fitting for the Py decay plots.

^†^Obtained from reactions for 2 h.

^‡^Reaction time, 12 h; +, yes; −, no.

^§^Note that kaolin consists largely of kaolinite.

^||^Acid-activated montmorillonite.

^¶^Acidic surface of ATD was pre-titrated with NH_3_. See Methods for details.

^#^As a control.
