# Supplementary material for: Mineral dust aerosols promote the formation of toxic nitropolycyclic aromatic compounds
Source: Sci Rep. 2016 Apr 14;6:24427. doi: 10.1038/srep24427 (PMC4830986; doi:10.1038/srep24427)
Supplement: Supplementary Information [file srep24427-s1.pdf]

[Supplementary Information]

Mineral dust aerosols promote the formation of toxic nitropolycyclic aromatic compounds

Takayuki Kameda,[1]* *Eri Azumi*,[2] *Aki Fukushima*,[2] *Ning Tang*,[2] *Atsushi Matsuki*,[3] *Yuta Kamiya*,[1] *Akira Toriba*,[2] *Kazuichi Hayakawa*[2,3]

[1] Graduate School of Energy Science, Kyoto University

Yoshida-Honmachi, Sakyo-ku, Kyoto 606-8501, Japan

Phone: +81-75-753-5621, Fax: +81-75-753-5619

[2] Institute of Medical, Pharmaceutical and Health Sciences, Kanazawa University

Kakuma-machi, Kanazawa, Ishikawa 920-1192, Japan

[3] Institute of Nature and Environmental Technology, Kanazawa University

Kakuma-machi, Kanazawa, Ishikawa 920-1192, Japan

*Corresponding author: tkameda@energy.kyoto-u.ac.jp (T. Kameda)

Supplementary Methods

Heterogeneous reaction of pyrene (Py) with NO_2

All the substrates used in this study were cleaned up by sonicating them twice in dichloromethane for 20 min before use. The surface of the substrates was coated with Py by a standard procedure based on liquid-solid adsorption, i.e. 10 mL of 30 μmol L^{-1} dichloromethane solution of Py was well mixed with 300 mg of a substrate in a Pyrex recovery flask, and then dichloromethane was gently removed by a rotary evaporator at 313 K under reduced pressure in the dark. When all the molecules of Py are adsorbed on the surface, the amount of supported Py per mg of the substrate is calculated to be 1 nmol mg^{-1}. The actual amount of the Py coated on each substrate was confirmed before the NO_2 exposure, i.e. the initially coated Py was extracted from a portion of the substrate sample and quantified by gas chromatographic-mass spectrometry (GC/MS) as described below. The actual initial load of Py in this study was typically 0.6 – 0.9 nmol mg^{-1}. In Fig. 1 and Supplementary Fig. S1, amounts of the reaction products and remaining Py are normalized to the initial load of Py on each substrate.

Py was heterogeneously reacted with gaseous NO_2 in a Pyrex cylindrical reaction vessel (6 cm ID × 18 cm height, ca. 500 mL) under ambient pressure at 298 ± 1 K and < 2% relative humidity in the dark (Supplementary Fig. S7). A portion of the Py-coated substrate (ca. 10 mg) was spread on a Pyrex petri dish (1.9 cm ID × 1.2 cm depth). The Petri dish was placed in the reactor and subsequently exposed to NO_2/air which was supplied by passing pure air through a permeation tube (Gastec, P-9-1) at a constant flow rate. Prior to the reaction, the inside of the reaction system was continuously exposed to 3 ppmv NO_2 for 24 h in order to minimize the loss of the reactant gas onto the walls of the system during the experiment. Different NO_2 mixing ratios ranging from 0.1 to 3 ppmv were obtained by changing the flow rate of the air from 6 to 0.4 L min^{-1}. When the flow rate was 0.4 L min^{-1}, a steady NO_2 concentration in the reactor was attained within 8 min. This was only a small part of the total reaction time (normally 1 – 12 h) so it shouldn't have appreciably affected the results. In order to clarify the loss of Py due to desorption and/or oxidation during the exposure, blank experiments on Chinese desert dust (CDD) were performed under the same conditions as those used for the reactions, but in the absence of NO_2. The observed loss rate of Py due to desorption/oxidation was very slow (less than one standard error of the Py degradation rate in the presence of 100 ppbv NO_2, Supplementary Fig. S1), and therefore was not expected to affect the kinetic results. The relative humidity and the NO_2 concentration in the reaction system were measured with a humidity probe (Vaisala, HMI41) and a chemiluminescence NOx analyzer (Thermo, Model 42s), respectively. Reaction products and the residual Py after the prescribed reaction time were extracted with dichloromethane as described below. The extracted chemicals were identified and quantified by GC/MS by comparing their retention times, fragmentation patterns, and peak areas with those of authentic standards.

Heterogeneous reaction of Py with HNO_3

The substrates used in this study were coated with Py by liquid-solid adsorption at a ratio of ~1 nmol mg^{-1}. Py was heterogeneously reacted with gaseous HNO_3 in a Pyrex cylindrical reaction vessel (6 cm ID × 18 cm height, ca. 500 mL) under ambient pressure at 298 ± 1 K and < 2% relative humidity in the dark. A portion of the Py-coated substrate (ca. 10 mg) was spread on a Pyrex petri dish (1.9 cm ID × 1.2 cm depth). The Petri dish was placed in the reactor and subsequently exposed to 2 ppmv HNO_3/air which was supplied by passing pure air through a permeation tube (KIN-TEK, 2022) at a constant flow rate of 0.5 L min^{-1}. Prior to the reaction, the inside of the reaction system was continuously exposed to 2 ppmv HNO_3 for 24 h in order to minimize the loss of the reactant gas onto the walls of the system during the experiment. Reaction products and the residual Py after the prescribed reaction time were extracted with dichloromethane as described below. The extracted chemicals were identified and quantified by GC/MS by comparing their retention times, fragmentation patterns, and peak areas with those of authentic standards.

Extraction of soluble organic fractions (SOF) from airborne particulate and laboratory experiment samples

The filter samples of ambient airborne particulates, onto which deuterated 1-nitropyrene (1-NP) and deuterated chrysene (1-NP-d_9 and Chry-d_{12}, respectively; internal standards) were added, were cut into fine pieces before extraction. SOF from the filter samples were extracted twice with 100 mL of dichloromethane under sonication for 20 min. The extract solution was filtered with a cellulose acetate filter to remove solid residue, followed by adding 100 μL of dimethyl sulfoxide (DMSO) into the filtrate to avoid complete dryness of the solvent during the concentration steps. After concentration to ca. 5 mL and filtration with a 0.45 μm membrane filter, the samples were concentrated to ca. 100 μL under a nitrogen stream to leave DMSO, and then 400 μL of methanol was added. An aliquot of each of the sample solution was subjected to the quantification of PAHs and 1-NP by high-performance liquid chromatograph (HPLC) analysis.

The NO_2 or HNO_3 exposure experiment products and the remaining reactant after each reaction were extracted with 10 mL of dichloromethane under sonication for 20 min after adding 1-NP-d_9 and deuterated pyrene (Py-d_{10}) as internal standards. The extract solution was filtered with a 0.45 μm membrane filter, and then concentrated to ca. 1 mL under a nitrogen stream.

Instrumentation for chromatographic analyses

An HPLC system with column-switching and chemiluminescence detection was employed for ambient particle-associated 1-NP quantification as reported previously.[1] Briefly, the system consists of four HPLC pumps, a 6-port switching valve, a clean up column (GL Sciences, Inertsil ODS-P, 3.0 mm ID × 250 mm), a separation column (GL Sciences, Inertsil ODS-3, 3.0 mm ID × 250 mm), a reducer column (Jasco, NPpak-RS, 4.0 mm ID × 10 mm), a trapping column (GL Sciences, Inertsil ODS-3, 4.0 mm ID × 30 mm), and a chemiluminescence detector (Soma Optics, S-3400). The chemiluminescence reagent solution was an acetonitrile solution containing 0.03 mmol L^{-1} bis(2,4,6-trichlorophenyl)oxalate and 15 mmol L^{-1} H_2O_2. Mobile phases were methanol/water (3/1, v/v) for the clean up and reduction of 1-NP, and acetonitrile/imidazole-perchloric acid buffer (45/55, v/v) for the separation. The reduction of 1-NP into 1-aminopyrene, which is strongly fluorescent, was performed at 373 K in the reducer column. HPLC analysis of the nine kinds of ambient particle-associated PAHs (Py, fluoranthene, chrysene, benz[*a*]anthracene, benzo[*b*]fluoranthene, benzo[*k*]fluoranthene, benzo[*a*]pyrene, dibenz[*a,h*]anthracene, and benzo[*ghi*]perylene) was performed with a Hitachi L-6200 pump, an F-1050 fluorescence detector, and an Inertsil ODS-3 column (3.0 mm ID × 250 mm, GL Sciences) as previously described.[2] Solutes were eluted isocratically using methanol/water (77/23, v/v) as the mobile phase at a flow rate of 0.45 mL min^{-1}. Quality assurance/quality control (QA/QC) of the analysis was evaluated by measuring parameters as recovery, linearity, sensitivity, repeatability and reproducibility. The recoveries of 1-NP and PAHs were more than 90%. Neither 1-NP nor PAHs were detected in the filter blank. Good linearity of calibration curves ($R^2 > 0.999$) was obtained when the amounts of the injected sample were in the range of 2 – 400 fmol for 1-NP and in the range of 0.5 – 50 pmol for PAHs. The limit of quantification for 1-NP was 2 fmol/injection (S/N = 10). The limits of quantification for PAHs were < 0.5 pmol/injection (S/N = 10). The precision and accuracy of the determination of 1-NP and PAHs were examined by adding known amounts of 1-NP and PAHs to the SOF extracted from airborne particulate samples. The relative standard deviations (RSD) of the intra- and inter-day precision study were within 10%. The accuracy values of the intra- and inter-day study were in the range of 92 – 101%. These values indicate that the methods used in this study are satisfactory for determining 1-NP and PAHs in airborne particulates.

GC/MS analysis for the reaction products and the residual reactant was performed using a mass spectrometer (Shimadzu, GC-2010 connected to QP-2010 Plus) with electron ionization (EI) method in the selected ion monitoring (SIM) mode; ionization voltage, 70 eV; interface temperature, 573 K; ion source temperature, 523 K. The GC column used was a ZB-5MS (5% phenylmethylsilicone, 0.25 mm ID × 30 m, Phenomenex). The temperature program for GC/MS analysis was as follows: 363 K for 2 min, to 593 K with ramping rate of 7 K min^{-1}, isothermal for 8 min. The monitored ions were m/z 202, 101 and 88 for Py, m/z 212 and 106 for Py-d_{10}, m/z 247, 217 and 201 for 1-NP, m/z 256, 226 and 210 for 1-NP-d_9, m/z 292, 262 and 200 for dinitropyrenes (DNPs).

Chemicals

1-NP, Chry-d_{12}, and Py were purchased from Wako Pure Chemical Industries. 1-NP-d_9 and Py-d_{10} were obtained from C/D/N Isotopes and Cambridge Isotope Laboratories, respectively. PAH standard mixture (EPA 610 Polynuclear Aromatic Hydrocarbons Mix) and DNPs were purchased from Supelco and Accu Standard, respectively. All solvents and other chemicals used were HPLC or analytical grades from Wako Pure Chemical Industries.

Supplementary Results

Estimation of fractional surface coverage of Py on ambient dust

It was reported that in Beijing, the surface area density of the atmospheric mineral dust was 6.3×10^{-6} cm^2 cm^{-3} when the dust concentration was 61 μg m^{-3}.[3] Hence, the specific surface area of the atmospheric dust is calculated to be 10 m^2 g^{-1}. Using the calculated specific surface area and the mean concentration of the atmospheric dust observed on 20 March, 2010 in Beijing (247 μg m^{-3}), the surface area density of the dust is estimated to have been 2.5×10^{-5} cm^2 cm^{-3} when the dust storm hit Beijing. Using the mean concentration of Py during 19 – 21 March, 2010 (8.6 pmol m^{-3}) and the effective cross-section of a Py molecule (~0.8 nm^2), the surface area density of Py molecules is calculated to have been 4.1×10^{-8} cm^2 cm^{-3}. Assuming that all the molecules of Py that we determined were adsorbed on the dust particles, thus, the fractional surface coverage of Py on the dust particles is estimated to have been 1.7×10^{-3}, i.e., the surface-adsorbed Py is believed to have formed a submonolayer on the dust particles.

Heterogeneous reaction of Py with HNO_3

Supplementary Fig. S6 shows time profiles of Py degradation and 1-NP formation under 2 ppmv HNO_3-air in the dark on CDD (a) and on ATD particles (b). The parent Py moderately decreased on both the substrates, and then ~30% of the initial Py was converted to 1-NP after 12 h of the reaction. No DNP was formed during the exposure. The apparent rate constants, k_{obs}, were $(3.6 \pm 0.1) \times 10^{-5}$ sec^{-1} and $(2.4 \pm 0.4) \times 10^{-5}$ sec^{-1} on CDD and ATD, respectively (errors represent one standard error). The k_{obs} values for the NO_2 reactions were an order of magnitude larger than those for the HNO_3 reactions. Concentration of NO_2 is typically 1 – 2 orders of magnitude larger than that of HNO_3 in urban air.[4] Therefore, the reaction of Py with HNO_3 appears to have little influence on the formation of 1-NP on dust particles.

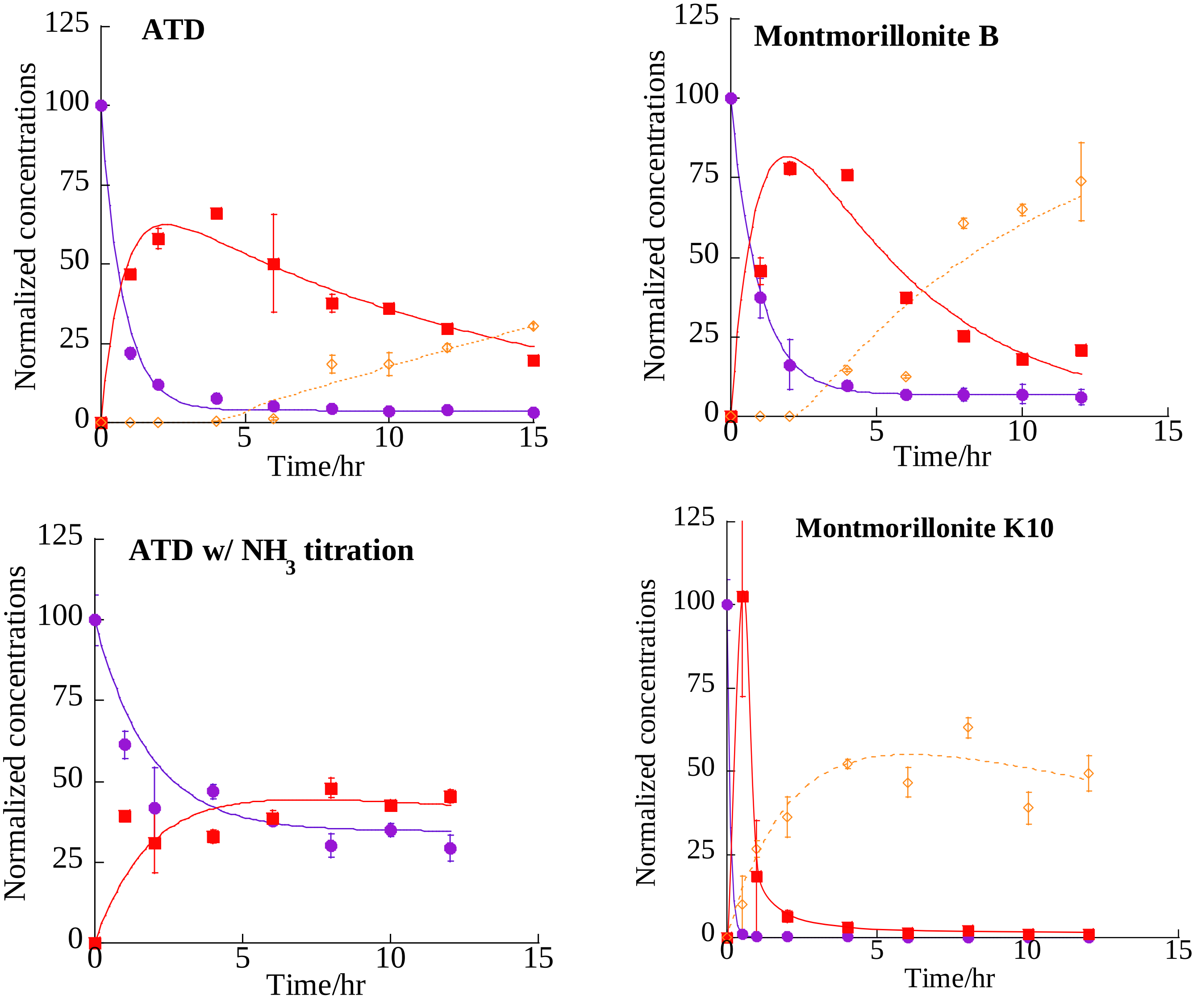

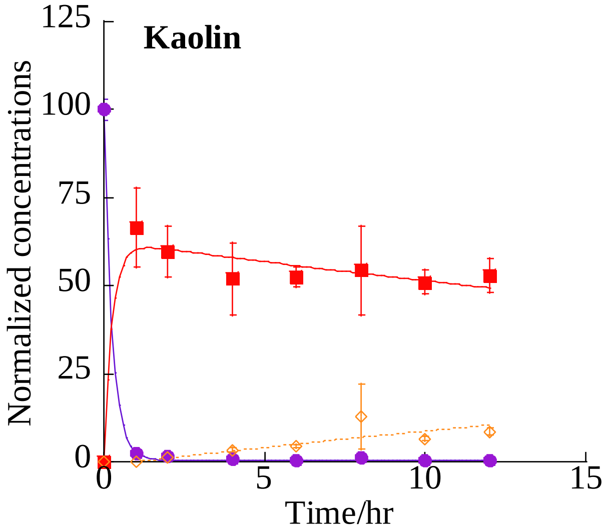

Supplementary Figure S1. Normalized concentrations of Py (circles) and nitropyrenes (1-NP, squares; DNPs, diamonds) on typical substrates after exposure to 3 ppmv NO_2 for the indicated times. The data points represent mean values (± 1 SD) of triplicate experiments. The curves for Py decay are exponential nonlinear least-squares fits assuming first-order reactions. The curves for nitropyrene formation are for illustrative purposes only.

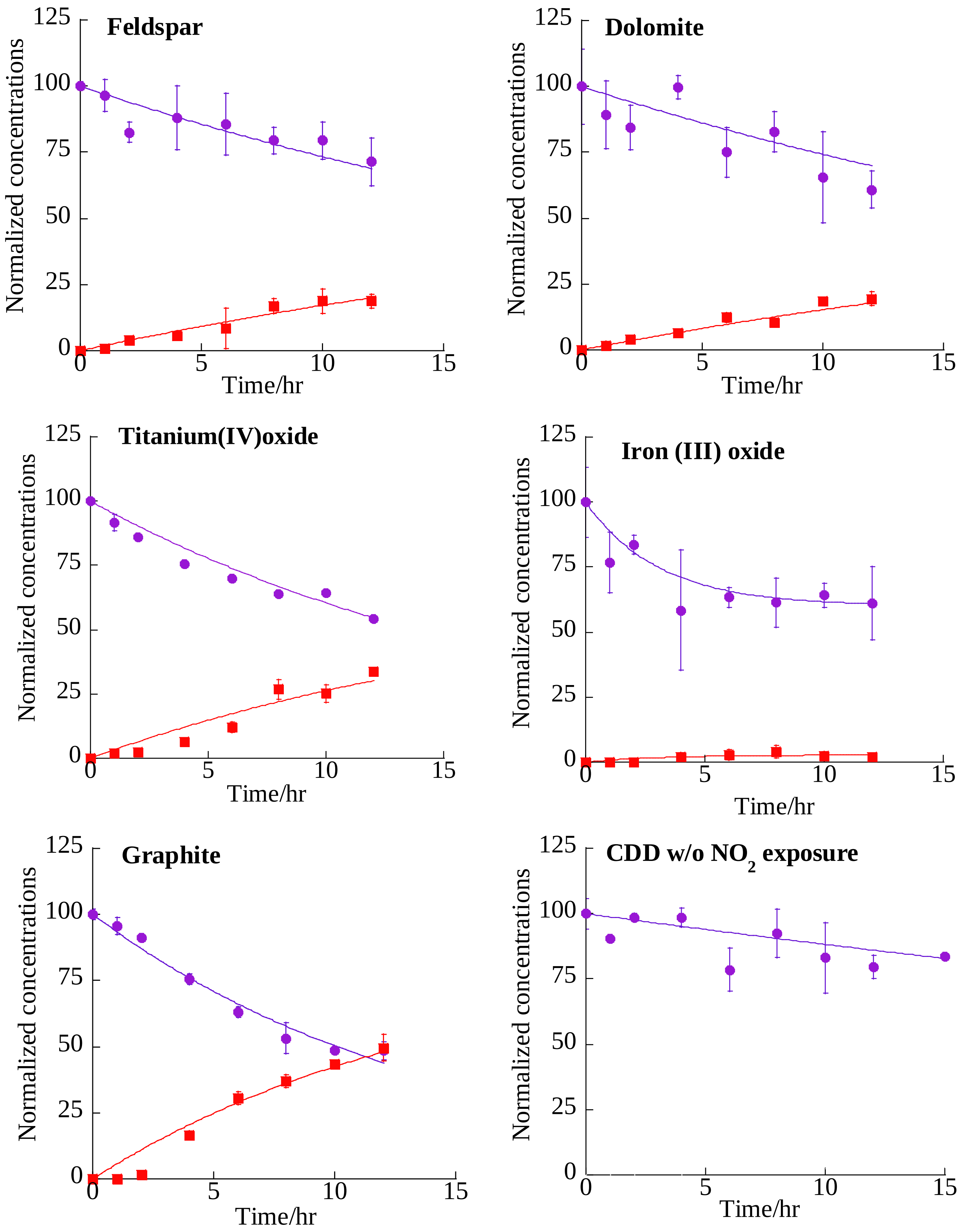

Supplementary Figure S1. Continued.

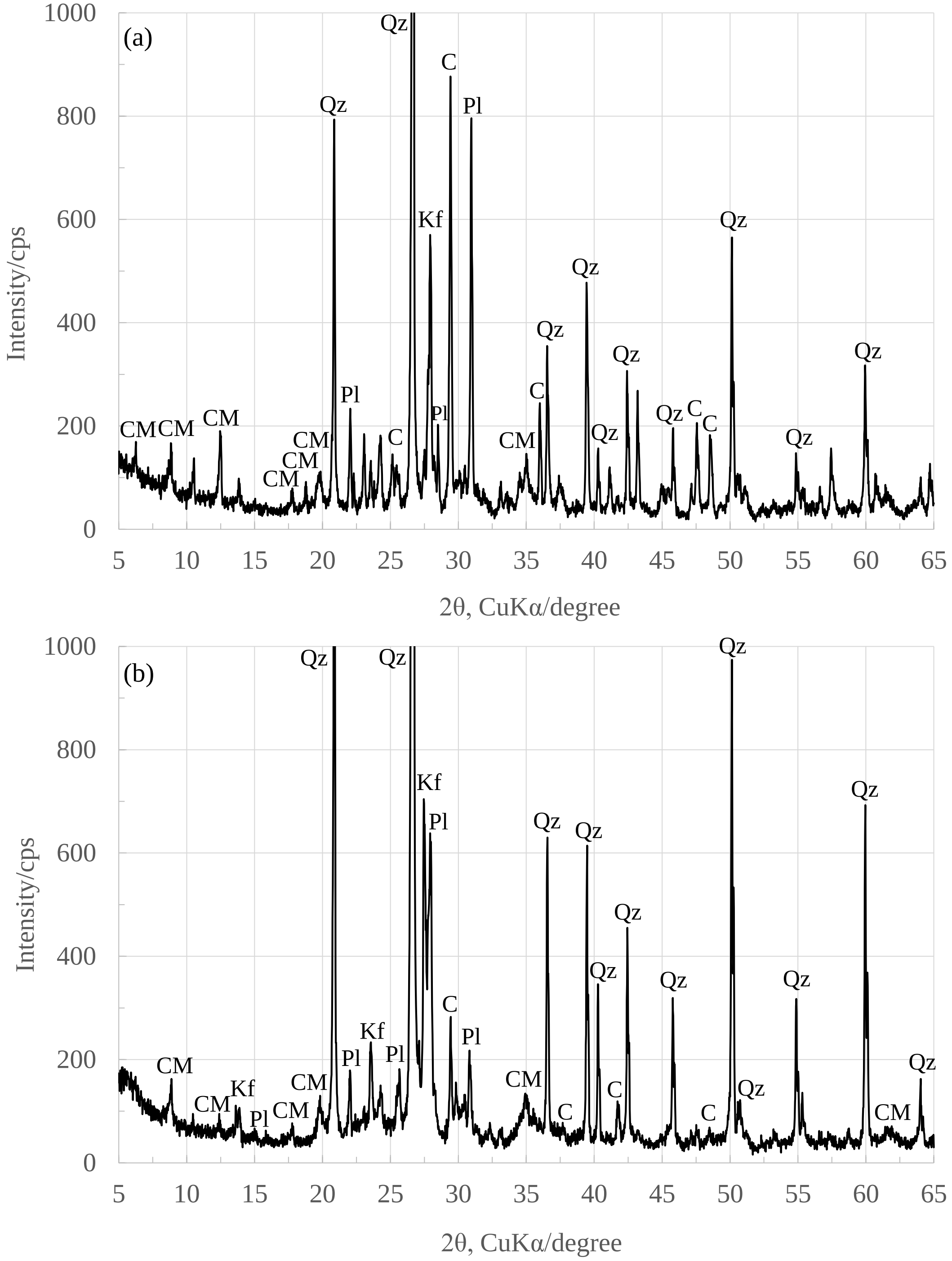

Supplementary Figure S2. XRD patterns for CDD and ATD. (**a**) CDD; (**b**) ATD. CM, clay minerals; Qz, quartz; Kf, potassium feldspar; Pl, plagioclase; C, calcite. Results are similar to those for Gobi and Tengger Deserts dust which were previously reported,[5] i.e., characteristic peaks for clay minerals as well as quartz, feldspars, and calcite were found.

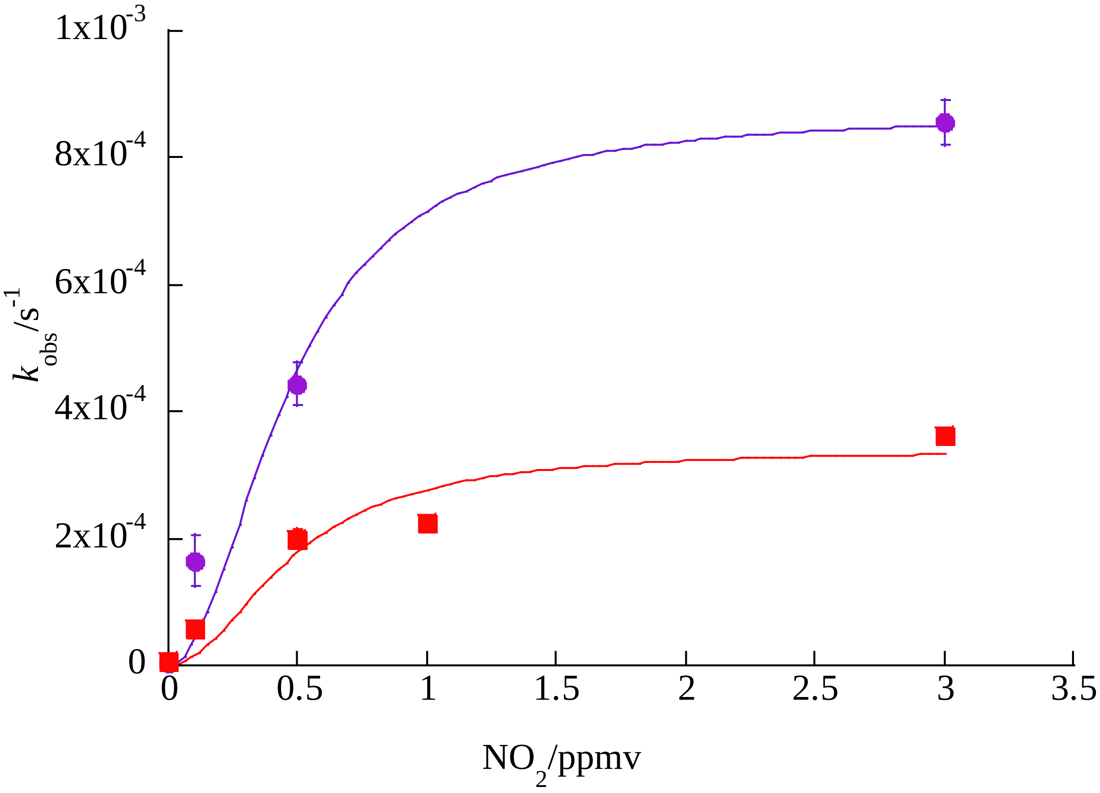

Supplementary Figure S3. Pseudo-first order rate coefficient (k_{obs}) as a function of gas-phase NO_2 concentration. The curves are nonlinear least-squares fits based on equation (4) in the main text. The upper data set was for CDD, the lower one for ATD. The error bars represent one standard error derived from nonlinear least-squares fitting for the Py decay plots.

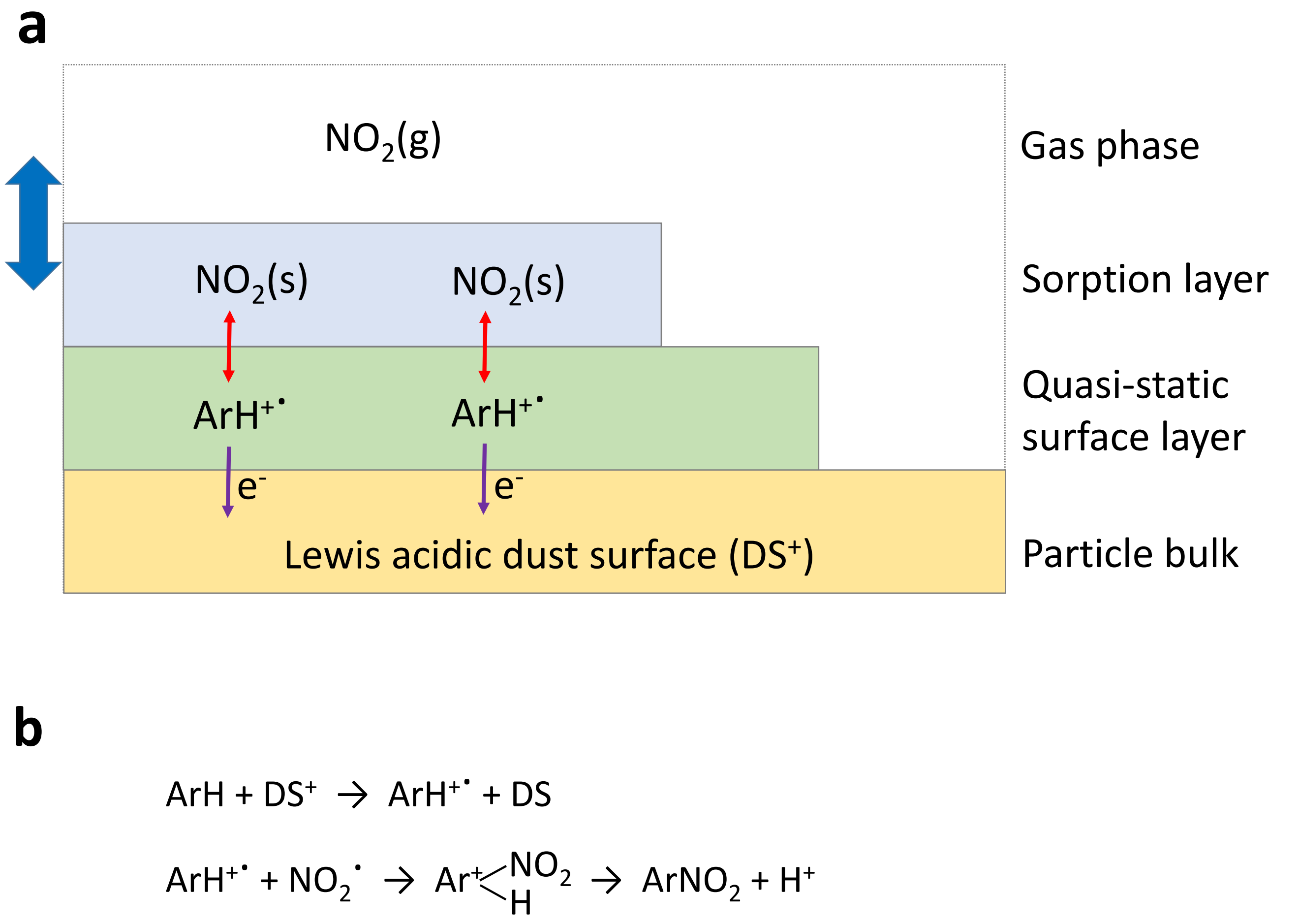

Supplementary Figure S4. Proposed mechanisms for the nitration of aromatic compounds (ArH) on the acidic surface of mineral dust. (a) Schematic illustration of the heterogeneous nitration. The gas-particle interface is divided into a gas-phase with gaseous NO_2 (NO_2(g)), a sorption layer with adsorbed NO_2 (NO_2(s)), a quasi-static surface layer with the aromatic radical cation ($ArH^{+\bullet}$), and a particle bulk. The blue arrow indicates the adsorption and desorption fluxes of NO_2. The red and purple arrows indicate chemical reactions and electron transfer, respectively. This heterogeneous chemistry is based on the Pöschl–Rudich–Ammann (PRA) framework.[6–8] **(b)** Chemical reaction scheme of the nitroaromatic compound ($ArNO_2$) formation on Lewis acid sites of the dust surface (DS^+).

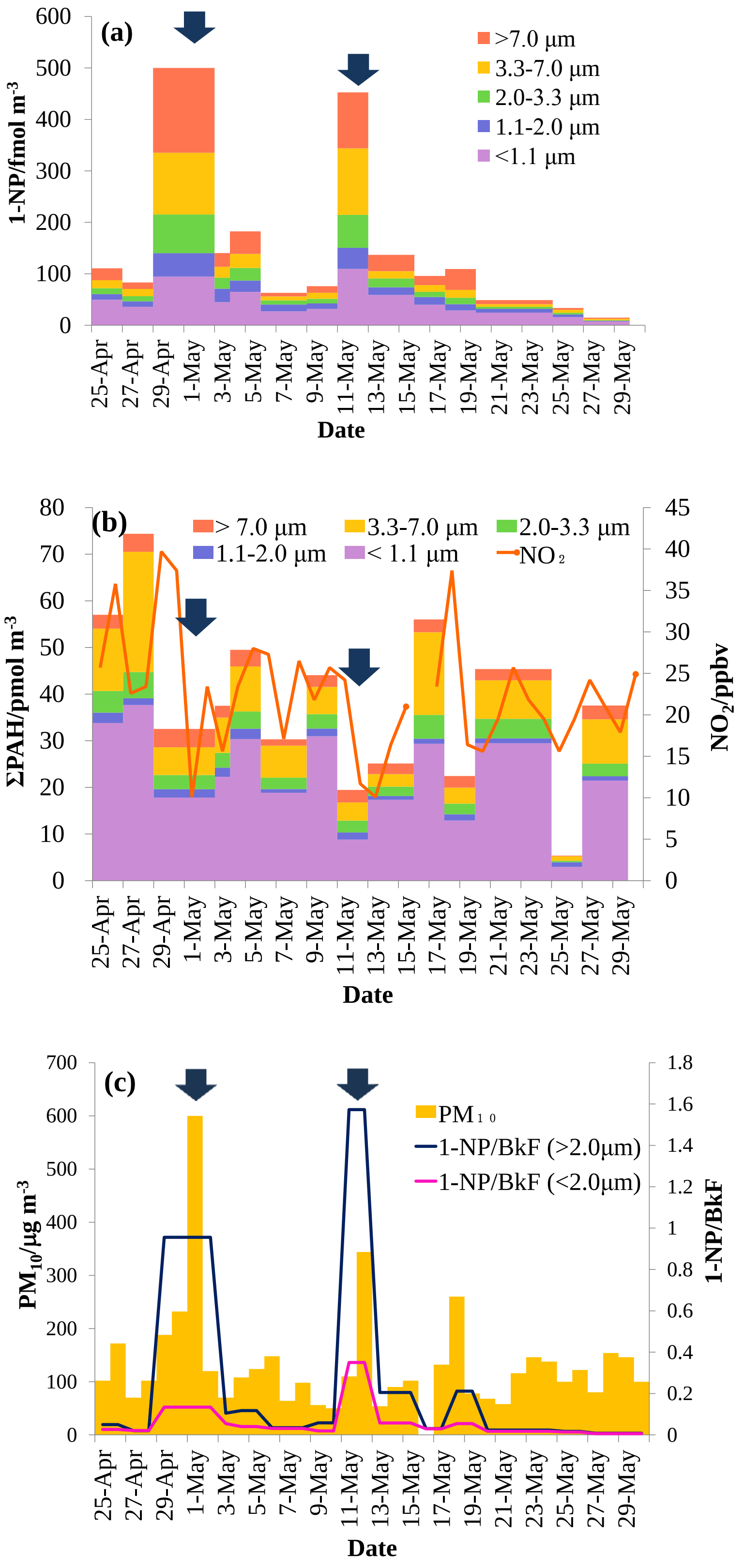

Supplementary Figure S5. Airborne particles (PM_{10}), PAHs, NO_2 and 1-NP concentrations in Beijing during 25 April – 30 May, 2011. (**a**) Size-fractionated particle-bound 1-NP. (**b**) Gaseous NO_2 and size-fractionated particle-bound PAHs. (**c**) PM_{10}. Variation in concentration of 1-NP relative to that of BkF (1-NP/BkF) is also shown in (c). Arrows indicate heavy dust periods (30 April – 1 May and 11 – 12 May).

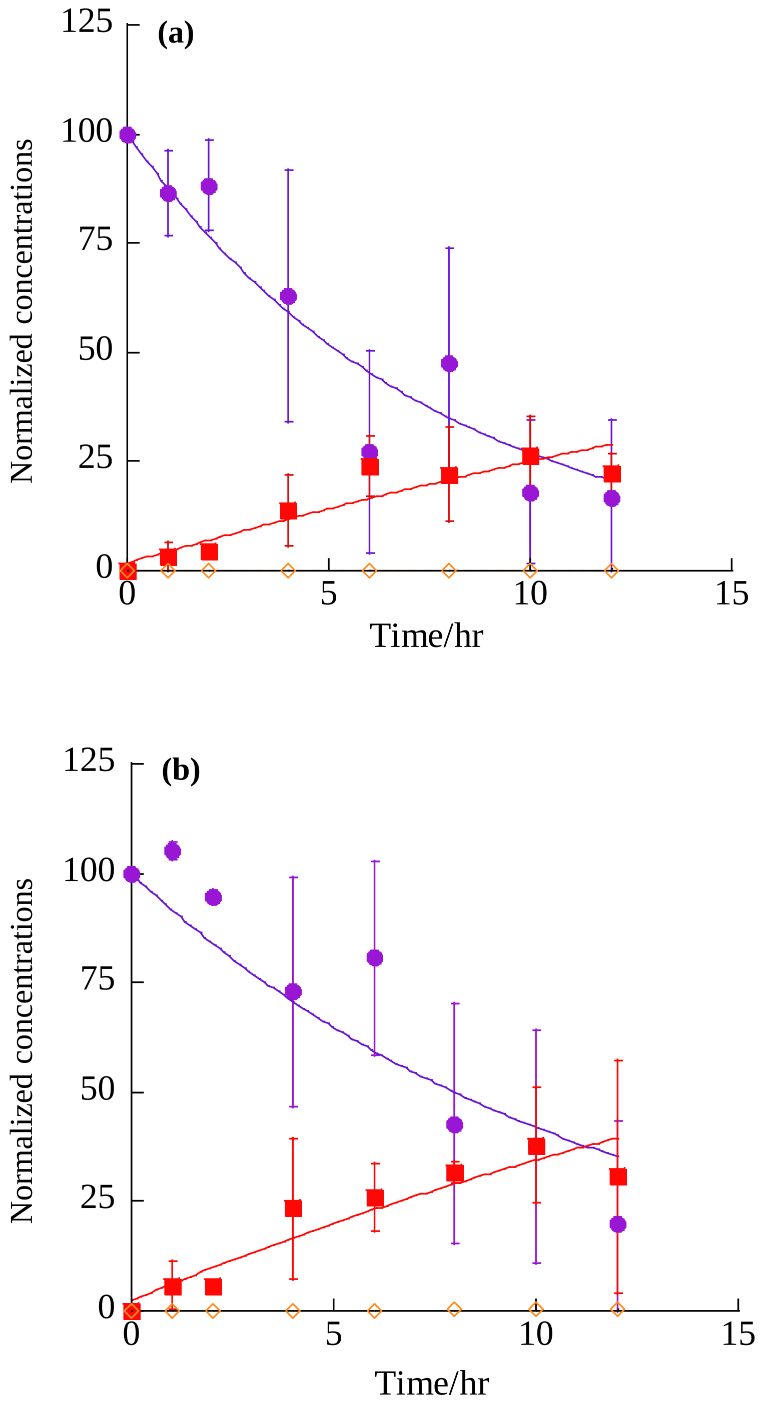

Supplementary Figure S6. Normalized concentrations of Py (circles) and nitropyrenes (1-NP, squares; DNPs, diamonds) on CDD (a) and ATD (b) after exposure to 2 ppmv HNO_3 for the indicated times. The data points represent mean values (± 1 SD) of triplicate experiments. The curves for Py decay are exponential nonlinear least-squares fits assuming first-order reactions. The curves for 1-NP formation are for illustrative purposes only.

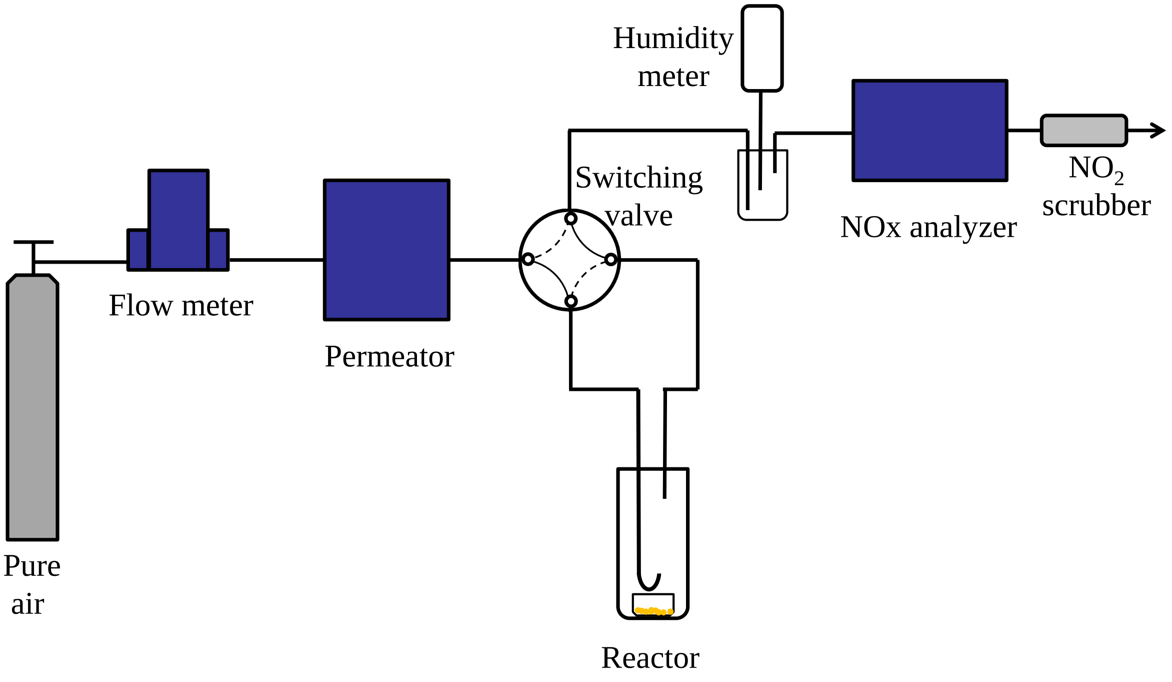

Supplementary Figure S7. Schematic diagram of the equipment for the heterogeneous reaction performed in this study.

Supplementary Table S1. Observed pseudo-first order rate constants for the reaction of Py on CDD and ATD with 0.1 – 1 ppmv NO_2 (k_{obs}), obtained adsorption equilibrium constants (K_{NO2}), and the maximum pseudo-first order rate constants (k_{max}). Errors represent one standard error derived from nonlinear least-squares fitting by equations (2) or (5).

Substrates	k_{obs}/10^{-3} s^{-1}			k_{max}/10^{-3} s^{-1}	K_{NO2}/10^{-13} cm^3
	$[NO_2]_g$ = 0.1 ppmv	$[NO_2]_g$ = 0.5 ppmv	$[NO_2]_g$ = 1 ppmv		
CDD	0.17 ± 0.04	0.44 ± 0.03	- *	1.0 ± 0.8	0.58 ± 0.11
ATD	0.059 ± 0.002	0.20 ± 0.02	0.22 ± 0.01	0.43 ± 0.01	0.58 ± 0.03

* No data

Supplementary Table S2. Sampling details for particulate samples in Beijing.

Sample ID	Date assignment in Fig. 4 and Fig. S5	Year	Start date	Start time	Stop date	Stop time	Total sample volume/m^3
2010-1	1-Mar - 2-Mar	2010	1-Mar	16:30	3-Mar	16:35	1627.9
2010-2	3-Mar - 4-Mar	2010	3-Mar	17:10	5-Mar	16:16	1699.6
2010-3	5-Mar - 7-Mar	2010	5-Mar	17:17	8-Mar	17:20	2445.3
2010-4	8-Mar - 9-Mar	2010	8-Mar	18:11	10-Mar	16:32	1623.2
2010-5	10-Mar - 11-Mar	2010	10-Mar	18:45	12-Mar	16:32	1535.1
2010-6	12-Mar - 16-Mar	2010	12-Mar	17:10	17-Mar	16:45	4057.3
2010-7	17-Mar - 18-Mar	2010	17-Mar	17:20	19-Mar	14:50	1546.3
2010-8	19-Mar - 21-Mar	2010	19-Mar	15:43	22-Mar	14:35	2412.1
2010-9	22-Mar - 23-Mar	2010	22-Mar	15:10	24-Mar	16:42	1677.4
2010-10	24-Mar - 25-Mar	2010	24-Mar	17:40	26-Mar	15:28	1556.6
2010-11	26-Mar - 28-Mar	2010	26-Mar	16:33	29-Mar	14:24	2360.0
2010-12	29-Mar - 30-Mar	2010	29-Mar	15:00	31-Mar	15:50	1659.2
2011-1	25-Apr - 26-Apr	2011	25-Apr	15:50	27-Apr	16:28	1678.8
2011-2	27-Apr - 28-Apr	2011	27-Apr	17:30	29-Apr	16:38	1614.6
2011-3	29-Apr - 2-May	2011	29-Apr	17:02	3-May	14:36	3172.4
2011-4	3-May	2011	3-May	15:07	4-May	17:15	886.6
2011-5	4-May - 5-May	2011	4-May	17:55	6-May	17:00	1596.2
2011-6	6-May - 8-May	2011	6-May	18:10	9-May	17:07	2408.0
2011-7	9-May - 10-May	2011	9-May	17:48	11-May	15:50	1555.4
2011-8	11-May - 12-May	2011	11-May	16:55	13-May	16:34	1624.0
2011-9	13-May - 15-May	2011	13-May	17:30	16-May	16:45	2416.9
2011-10	16-May - 17-May	2011	16-May	17:25	18-May	15:52	1571.7
2011-11	18-May - 19-May	2011	18-May	16:22	20-May	19:36	1736.1
2011-12	20-May - 24-May	2011	20-May	20:30	25-May	16:09	3927.4
2011-13	25-May - 26-May	2011	25-May	16:50	27-May	14:08	1538.8
2011-14	27-May - 29-May	2011	27-May	15:10	30-May	15:17	2446.8

Supplementary Table S3. Chemical composition (wt %) of mineral substrates examined in this study.

Substrates	SiO_2	Al_2O_3	Fe_2O_3	TiO_2	MnO	MgO	CaO	Na_2O	K_2O	P_2O_5
Limestone *	0.12	0.02	0.02	< 0.01	< 0.01	0.61	55.1	< 0.01	< 0.01	0.03
Dolomite *	0.22	0.02	0.02	< 0.01	< 0.01	18.5	34.0	0.01	< 0.01	0.03
Feldspar *	66.7	18.1	0.06	0.005	< 0.01	< 0.01	0.93	3.37	9.99	0.01
Sodium feldspar †	67.7	20.0	0.06	0.03	< 0.01	0.10	0.55	11.3	0.14	0.14
Potassium feldspar †	67.0	18.0	0.08	0.01	< 0.01	0.04	0.10	3.32	11.0	< 0.01
CDD	57.7	9.26	4.73	0.77	0.09	3.82	11.6	1.69	1.8	
ATD ‡	68-76	10-15	2-5	0.5-1.0		1-2	2-5	2-4	2-5	
Kaolin †	49.8	35.6	0.28	0.07		< 0.01	< 0.01	0.03	< 0.01	0.11
Montmorillonite A §	66.4	11.9	1.6	0.1		2.6	0.5	2.0	1.3	0.01
Montmorillonite B §	54.0	19.9	1.9	0.1		3.0	0.4	3.4	0.4	0.01
Montmorillonite K10 ‖	73.0	14.0	2.7			1.1	0.2	0.6	1.9	
Saponite §	45.8	4.4	0.0	0.0		25.6	0.1	3.2	0.1	0.00

* Certified by the Geological Survey of Japan.

† Certified by the Ceramic Society of Japan.

‡ Provided by the supplier.

§ Taken from Supplementary Reference 9.

‖ Taken from Supplementary Reference 10.

Supplementary Table S4. Specific surface area and mean diameter of the substrates used in this study.

Substrates	BET surface area/m^2 g^{-1}	* Mean diameter/μm
CDD	18.2	21.53
ATD	7.4	12.57
Kaolin	25.9	21.46
Montmorillonite A	27.4 ‡	3.31 ‡
Montmorillonite B	7.1 ‡	0.88 ‡
Saponite	105 ‡	0.03 ‡
Montmorillonite K10	250 §	- ‖
Pottasium feldspar	4.0	7.01
Sodium feldspar	0.7	26.75
Feldspar	1.4	22.24
Limestone	2.0	12.74
Dolomite	3.7	15.07
Calcium sulfate	0.6	25.83
Quartz	0.7	11.97
Aluminum oxide	7.8 - 9.2 §	0.35 - 0.49 §
Iron (III) oxide	5.8	22.94
Titanium (IV) oxide	10.5 §	0.33 §
Graphite	19.7	13.65

* On a mass basis.

‡ Taken from Supplementary Reference 9.

§ Provided by suppliers.

‖ No data.

Supplementary References

1 Kameda, T., Akiyama, A., Toriba, A., Tang, N. & Hayakawa, K. Atmospheric Formation of Hydroxynitropyrenes from a Photochemical Reaction of Particle-Associated 1-Nitropyrene. *Environ. Sci. Technol.* **45**, 3325-3332 (2011).

2 Kameda, T., Goto, T., Toriba, A., Tang, N. & Hayakawa, K. Determination of airborne particle-associated benz[*a*]anthracene-7,12-quinone using high-performance liquid chromatography with in-line reduction and fluorescence detection. *J. Chromatogr. A* **1216**, 6758-6761 (2009).

3 Huang, L., Zhao, Y., Li, H. & Chen, Z. Kinetics of heterogeneous reaction of sulfur dioxide on authentic mineral dust: effects of relative humidity and hydrogen peroxide. *Environ. Sci. Technol.* **49**, 10797-10805 (2015).

4 Kurita, K. & Aoki, K. Measurement of gaseous nitric acid and hydrochloric acid in the atmosphere using a continuous concentrator. *Annual report of the Tokyo Metropolitan Research Institute for Environmental Protection*, 308-312 (1992).

5 Nishikawa, M. *et al.* Preparation and chemical characterisation of an Asian mineral dust certified reference material. *Anal. Methods* **5**, 4088-4095 (2013).

6 Shiraiwa, M., Garland, R. M. & Pöschl, U. Kinetic double-layer model of aerosol surface chemistry and gas-particle interactions (K2-SURF): Degradation of polycyclic aromatic hydrocarbons exposed to O_3, NO_2, H_2O, OH and NO_3. *Atmos. Chem. Phys.* **9**, 9571-9586 (2009).

7 Springmann, M., Knopf, D. A. & Riemer, N. Detailed heterogeneous chemistry in an urban plume box model: reversible co-adsorption of O_3, NO_2, and H_2O on soot coated with benzo[*a*]pyrene. *Atmos. Chem. Phys.* **9**, 7461-7479 (2009).

8 Pöschl, U., Rudich, Y. & Ammann, M. Kinetic model framework for aerosol and cloud surface chemistry and gas-particle interactions - Part 1: General equations, parameters, and terminology. *Atmos. Chem. Phys.* **7**, 5989-6023 (2007).

9 Miyawaki, R. *et al.* Some Reference Data for the JCSS Clay Specimens. *Journal of the Clay Science Society of Japan* **48**, 158-198 (2010).

10 Flessner, U. *et al.* A study of the surface acidity of acid-treated montmorillonite clay catalysts. *J. Mol. Catal. A-Chem.* **168**, 247-256 (2001).
